# Thioredoxin reductase is a key factor in the oxidative stress response of *Lactobacillus plantarum *WCFS1

**DOI:** 10.1186/1475-2859-6-29

**Published:** 2007-08-28

**Authors:** L Mariela Serrano, Douwe Molenaar, Michiel Wels, Bas Teusink, Peter A Bron, Willem M de Vos, Eddy J Smid

**Affiliations:** 1 Top Institute Food and Nutrition, formerly WCFS, Wageningen, The Netherlands; 2 NIZO Food Research B.V., Ede, The Netherlands; 3 Wageningen UR, Laboratory of Microbiology, Wageningen, The Netherlands

## Abstract

**Background:**

Thioredoxin (TRX) is a powerful disulfide oxido-reductase that catalyzes a wide spectrum of redox reactions in the cell. The aim of this study is to elucidate the role of the TRX system in the oxidative stress response in *Lactobacillus plantarum *WCFS1.

**Results:**

We have identified the *trxB1*-encoded thioredoxin reductase (TR) as a key enzyme in the oxidative stress response of *Lactobacillus plantarum *WCFS1.

Overexpression of the *trxB1 *gene resulted in a 3-fold higher TR activity in comparison to the wild-type strain. Subsequently, higher TR activity was associated with an increased resistance towards oxidative stress. We further determined the global transcriptional response to hydrogen peroxide stress in the *trxB1*-overexpression and wild-type strains grown in continuous cultures. Hydrogen peroxide stress and overproduction of TR collectively resulted in the up-regulation of 267 genes. Additionally, gene expression profiling showed significant differential expression of 27 genes in the *trxB1*-overexpression strain. Over expression of *trxB1 *was found to activate genes associated with DNA repair and stress mechanisms as well as genes associated with the activity of biosynthetic pathways for purine and sulfur-containing amino acids. A total of 16 genes showed a response to both TR overproduction and hydrogen peroxide stress. These genes are involved in the purine metabolism, energy metabolism (*gapB*) as well as in stress-response (*groEL*, *npr2*), and manganese transport (*mntH2*).

**Conclusion:**

Based on our findings we propose that overproduction of the *trxB1*-encoded TR in *L. plantarum *improves tolerance towards oxidative stress. This response coincides with simultaneous induction of a group of 16 transcripts of genes. Within this group of genes, most are associated with oxidative stress response. The obtained crossover between datasets may explain the phenotype of the *trxB1*-overexpression strain, which appears to be prepared for encountering oxidative stress. This latter property can be used for engineering robustness towards oxidative stress in industrial strains of *L. plantarum*.

## Background

TRX was first characterized as a sole electron donor for ribonucleotide reductase in *Escherichia coli *[1]. The catalytic activity of these oxido-reductases can be attributed to the -CXXC- motif found in these proteins. At this cysteine-rich site electrons are transferred from the reduced TRX towards the substrate (proteins, disulfides, etc). The resulting oxidized TRX is regenerated via thioredoxin reductase (TR) using NADPH as a cofactor. Throughout the years, studies on the effect of the ubiquitous and conserved TRX in cellular metabolism have revealed that it plays a significant role in a variety of processes, including oxidative stress, protein repair, and RNA biosynthesis [2-4].

Intensive research on the role of TRX and TR include the use of transcriptomics and proteomics approaches. Studies with *Bacillus subtilis *and *Oenococcus oeni *showed that gene *trxA *was induced under stress conditions like heat and hydrogen peroxide stress [5,6]. In addition, gene expression studies in *E. coli *elucidated that under hydrogen peroxide stress, OxyR (transcription regulator activated by hydrogen peroxide in the cell) exerts transcription regulation on the TRX system [3]. Proteomic studies in microorganisms have allowed the identification of a range of TRX-targeted proteins via the use of a Tandem Affinity Purification tag. In addition, the *in-vivo *TRX-interacting proteins in *Saccaromyces cerevisiae *were identified using yeast two-hybrid systems [7]. Both proteomic approaches revealed possible associations within the complex networks of redox regulation in microorganisms and TRX and underlines the broad impact of the TRX system in the metabolic and regulatory network of the cell.

The reducing power of the TRX system and the glutaredoxin system is essential for all organisms. Furthermore, these two systems are suggested to be the only systems that maintain the cytoplasm of the cell in the reduced state [8]. While glutathione is found in eukaryotes and Gram-negative bacteria, it has been reported that most Gram-positive bacteria lack the ability to synthesize glutathione but rather import it from the environment. This is the case for *B. subtilis*, *Listeria monocytogenes*, and *Lactobacillus plantarum *[9]. In the latter organism the gene *gshB *which is involved in the second step for the synthesis of glutathione (glutathione synthase), has not been identified in the genome sequence [10]. Hence, it is believed that in *L. plantarum *the TRX system is the only active thiol-reducing system.

Little is known about the TRX system and its function in *L. plantarum*. This flexible and versatile bacterium is a member of the human gut microbiota, and is commonly used in fermented foods. The purpose of this study is to characterize the TRX system in *L. plantarum *WCFS1 pursuing a functional genomics approach where transcriptomics, enzyme activity assays, and bioinformatics studies will be used. This investigation clarifies the suggested roles of TR in the response of *L. plantarum *WCFS1 to oxidative stress. Finally, this study unveils the role of TRX reductase or the TRX system as a redox sensor in the cell.

## Results

### *In silico *analysis of the TRX system

The annotated genome of *L. plantarum *WCFS1 [10] reveals that the TRX system in this organism is composed by six ORF's (Open Reading Frames). Four genes are annotated as TRX encoding genes: *trxA1 *(lp_0236), *trxA2 *(lp_2270), *trxA3 *(lp_3437), and *trxH *(lp_2633). The other two genes *trxB1 *(lp_0761) and *trxB2 *(lp_2585) are annotated respectively as TR and nucleotide-disulphide oxidoreductase. These six genes are dispersed throughout the genome and are highly conserved within *L. plantarum *strains regardless of their ecological niche [11].

Bioinformatics tools were used to investigate the evolutionary relationship of the TRX genes in *L. plantarum *WCFS1. The translated gene sequences for *trxA2 *and *trxB1 *from *L. plantarum *WCFS1 showed the highest homology (p_scores _higher than e^-152^) to characterized TRX and TR respectively of different organisms such as *L. lactis *and *B. subtilis*. In *L. plantarum trxB1 *and *trxB2 *have a 28% similarity at the protein level. The orthologous relation of *trxB1 *from *L. plantarum *WCFS1 with *trxB *of *B. subtilis *and the similarity in the alignment with other *trxB *genes (including *trxB *of *E. coli*) suggests a molecular function of *trxB1 *as TR. Interestingly, the sequence of *trxB2 *does not possess an active center -CACV- which is an essential characteristic of the TR suggesting that this ORF does not have the potential of reducing TRX. By phylogeny analysis it was determined that the *trxB2*- encoding protein of *L. plantarum *WFCS1 is orthologous to the *B. subtilis *protein YUMC suggesting that these two sequences have the same molecular function namely to act as a ferredoxin NAD(P) reductase [12].

### *In vivo *functionality of the TRX system

The *in vivo *functionality of the complete TRX system was studied by quantitative PCR (q-PCR) of RNA isolated from *L. plantarum *WCFS1 grown in CDM at 37°C under anaerobic and oxidative stress conditions. The cultures were exposed to different temperatures or redox environments created by the addition of diamide or DTT to the medium. The relative expression expressed in arbitrary units (Au) of the six different transcripts that compose the TRX system is presented in Table 1. We observed that *trxA2 *and *trxB1 *were relatively higher expressed under all studied conditions when compared to the other *trxA's *and *trxB2 *respectively. Moreover, transcript *trxB2 *and *trxA2 *were respectively higher expressed when the bacterium was cultivated at 37°C compared to the other four studied transcripts evaluated at the same incubation temperature. When comparing the effect of different growth conditions within each probe, we observed -with the exception of *trxB2*- that the highest expression of each studied transcript is observed under diamide exposure compared to when grown with DTT or different temperatures (37°C and 30°C). Our observations suggest a role for both *trxA2 *and *trxB1 *genes in the response mechanism of this bacterium towards oxidative stress while the gene, *trxB2 *together with *trxA2 *could play a role during a heat shock or reductive stress.

**Table 1:** Relative expression levels of *trxA1*, *trxA2*, *trxA3*, *trxH*, *trxB1*, and *trxB2 *in *Lactobacillus plantarum *grown under different oxidative environments.

	***trxA1***		***trxA2***		***trxA3***		***trxH***		***trxB1***		***trxB2***	
	
Sample	*AV*^5^	*stdev*	*AV*^5^	*stdev*	*AV*^5^	*stdev*	*AV*^5^	*stdev*	*AV*^5^	*stdev*	*AV*^5^	*stdev*
A^1^	115,95	67	186,78	28	128,83	33	94,80	32	197,93	12	151,40	27
B^2^	98,18	66	506,75	23	122,20	7	132,15	33	367,80	47	392,55	14
C^3^	156,50	33	357,43	68	152,20	41	138,13	18	180,75	55	304,17	69
D^4^	240,25	30	2104,90	124	870,00	8	380,05	81	1258,68	102	139,43	65

^1^RNA extracted at OD_600 _= 1.5 from *L. plantarum *WCFS1 grown at 30°C on CDM with 0.5% Glucose

^2^RNA extracted from *L. plantarum *WCFS1 extracted at OD_600 _= 1.5 when grown at 37°C on CDM containing 0.5% Glucose.

^3^RNA isolated at OD_600 _= 1.5 from *L. plantarum *WCFS1 grown at 37°C in CDM containing 0.5% Glucose until OD_600 _= 1.0 when diamide was added to a final concentration of 5 mM

^4^RNA isolated at OD_600 _= 1.5 from *L. plantarum *WCFS1 grown at 37°C on CDM with 0.5% Glucose until OD_600 _= 1.0 when DTT was added to a final concentration of 5 mM

Based on the gene expression data and the *in silico *sequence analysis; we propose that *trxB1 *is coding for the main TR in the TRX system in *L. plantarum *WCFS1. To study the role of this enzyme in more detail, we constructed *L. plantarum *strains with elevated expression levels of *trxB1*.

### Overproduction of TR

**Figure 1:**
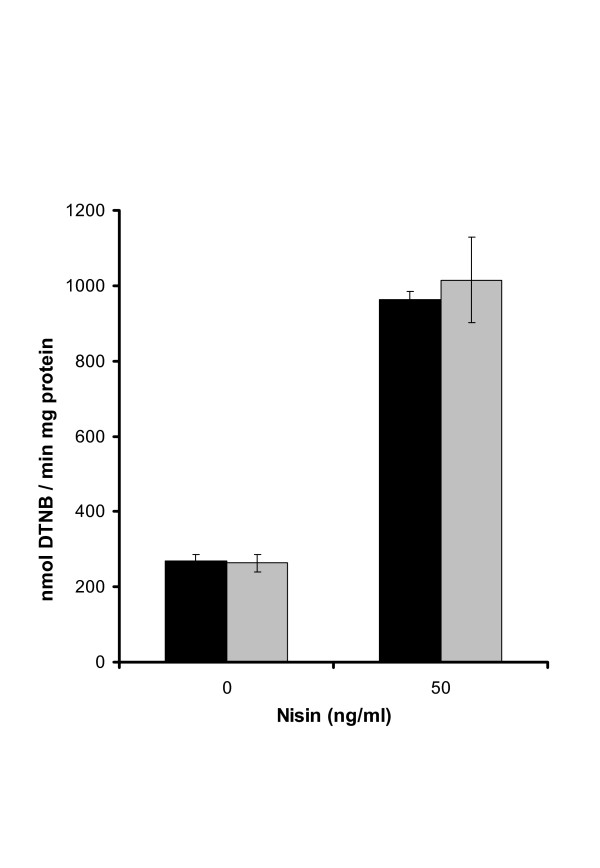
Thioredoxin reductase enzyme activity measurements showed as nmolDTNB reduced per min per mg protein. The strain NZ7601 was grown (black bars) aerobically and (gray bars) anaerobically at 37°C and was induced with 0 or 50 ng/ml nisinZ. After 5 hours of induction cell free extracts were prepared and TR activity was measured. The data shown above is the result of three independent experiments.

Overproduction of TR was achieved using the NICE expression system [13]. The transformant (*L. plantarum *NZ7601), carries the gene *trxB1 *under the control of the nisin promoter. Strain NZ7601 displayed five-fold higher TR activity after induction with 50 ng/ml nisinZ compared to the wild type strain NZ7606 (data not shown). The strain NZ7601 is able to reduce more than 1000 nmol DTNB·(min·mg prot)^-1 ^compared to 300 nmol DTNB·(min·mg prot)^-1 ^when no nisin is added to the medium (Fig. 1). The TR activity of NZ7601 without the addition of nisin corresponded to the TR activity of the control strain NZ7606 with values, both under aerobic and anaerobic growth conditions, of 300 and 320 nmol DTNB·(min·mg prot)^-1 ^respectively. An elevated expression of the *trxB1 *transcript was also detected in NZ7601 by northern blot analysis (data not shown).

**Figure 2:**
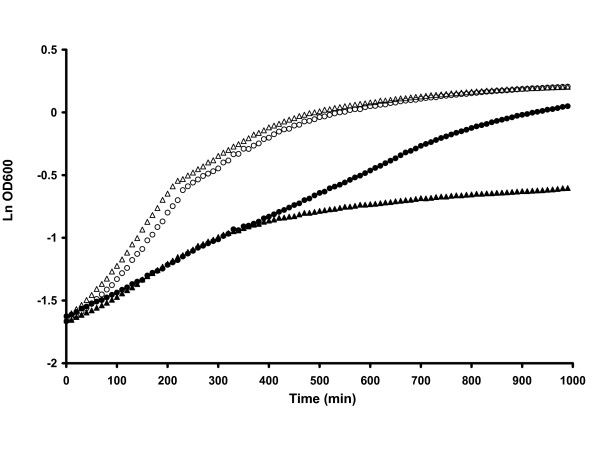
Growth patterns of nisin inducible *L. plantarum *NZ7100 strains on MRS supplemented with 10 μg/μl of chloramphenicol with or without diamide after nisin induction. The strain NZ7601 carrying the over expression of gene *trxB1 *was grown in MRS broth (white circles) and in MRS with 5 mM diamide (black circles) and the wild type strain NZ7606 was grown in MRS broth (white triangles) and in MRS with 5 mM diamide (black triangles). The growth was monitored by turbidity measurements at 600 nm.

Next, we evaluated the effect of overproduction of the reductase in the oxidative stress response. For this we monitored the growth of the *trxB1*-overexpressing strain NZ7601 and wild-type NZ7606 in the presence of diamide [5 mM], a known thiol oxidant [14] (Fig. 2). Both tested strains grew with a maximum specific growth rate (μ_max_) of 0.36 h^-1 ^when no stress factor was present in the medium. In the presence of diamide, both strains were initially similarly affected by the oxidative stress showing a μ_max _reduction to 0.17 h^-1 ^and 0.15 h^-1^, respectively. However, later on the growth pattern strain NZ7601 differed from that of the wild-type. While strain NZ7606 after reaching an OD_600 _of 0.5 stops growing and enters stationary phase, strain NZ7601 continues to grow under oxidative stress and reaches a cell density comparable to that observed in the absence of oxidative stress.

**Figure 3:**
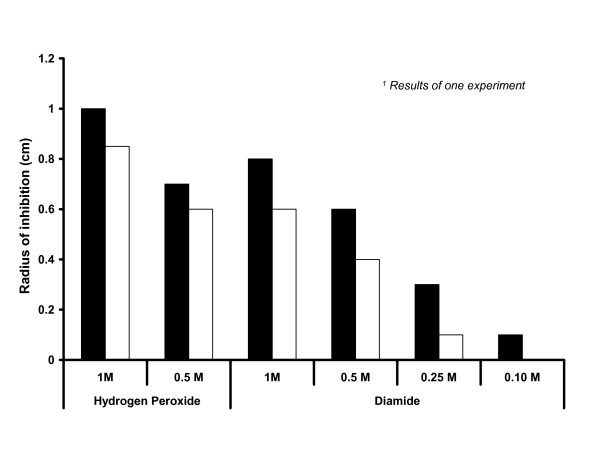
Growth-zone inhibition assays. The zone of growth inhibition towards hydrogen peroxide or diamide is showed in centimeters in the y-axis. The different strains are represented in the figure as such: *L. plantarum *NZ7606 (white bars) and *L. plantarum *NZ7601 (black bars).

This response under oxidative stress of strain NZ7601 was investigated in a quantitative growth-zone inhibition assay (Fig. 3). In this assay, we tested the inhibitory capacity of hydrogen peroxide and diamide on strains NZ7601 and NZ7606. We observed that strain NZ7601 at all studied oxidant concentrations showed a smaller inhibition zone compared to NZ7606.

### Transcriptome analysis

To analyze the correlation between TR overproduction and oxidative stress we carried out transcriptome analyses of the constructed strains. The mutant and the wild-type strains were grown in chemostats at a dilution rate of 0.1 h^-1^, and samples for transcriptome analysis were drawn at steady state both prior and 30 min after a hydrogen peroxide pulse. Because the NICE system is not suitable for continuous cultivations due to the instability of nisin in the medium (data not shown), we used a constitutive promoter, P_pepN_, to drive expression of *trxB1*. Therefore, two new strains were constructed: strain NZ7602 carrying plasmid pMS040 where *trxB1 *is under the control of the a constitutive promoter from *L. lactis *[15], P_pepN_, and strain NZ7607 as a control strain carrying the empty plasmid pNZ7021. The continuous cultivations were performed in biological triplicates (Table 2). In the chemostat cultivations glucose present in the medium (100 mM) was consumed to undetectable levels (<0.5 mM) and the biomass produced was comparable in both strains. In addition, we found no growth difference between both strains under this cultivation condition.

**Table 2:** Summary of parameters retrieved from continuous cultivations of *trxB1* over-expression strain *L. plantarum *NZ7602 and control strain *L. plantarum *NZ7607.

**Strain**	**Substrate**	**Physiological parameters**	
	**Glc^a^**	**Y_glc-X_^b^**	**GAPDH^c^**

Medium	100		
NZ7607	u.d	0.12	0.623 ± 0.02
NZ7602	u.d	0.13	1.613 ± 0.12
NZ7607 + perox	u.d	0.14	n.d
NZ7602 + perox	u.d	0.15	n.d

^a^mM of glucose present in the supernatant

^b^biomass yield on glucose (g of biomass/g glucose consumed).

^c^enzyme activity expressed as nmoles NADPH·(min·mg prot)^-1^

u.d. < 0.1 mM

n.d not determined

Mean ± SD of two separate chemostat steady states

The overexpression of *trxB1 *present in strain NZ7602 was in itself a good internal control in the transcriptome analysis. The transcript of this gene was found to be 23-fold more abundant in the *trxB1 *over-expressing strain compared to the control strain. This increase in transcript level resulted in a doubling of TR activity in strain NZ7602 compared to the wild-type (data not shown). The ANOVA statistical test that summarizes the significant effects due to overexpression of *trxB1 *showed that there were in total 27 significantly affected transcripts (p_value _< 0.01 and FC ≥1.5) in the *trxB1 *over-expressing strain when compared to the wild type (Table 3). We observed that 15% of them are predicted to be involved in the purine and pyrimidine biosynthesis. Other affected transcripts are predicted to be involved in stress-related processes (*groEL*, *npr2*), transport and binding proteins (*mntH2*), protein synthesis (*tuf*), protein fate (lp_1023), and genes involved in the cellular envelope (*ica3*). It is to be noted that none of the mentioned stress-related genes has a -CXXC- cysteine-rich active center. We also observed a significant up-regulation (1.7 fold) of the gene coding for glyceraldehyde-3-phosphate dehydrogenase, *gapB*. The GAPDH protein is involved in the energy metabolism of the cell. In *L. plantarum *WCFS1, *gapB*, is the only gene annotated as a glyceraldehyde-3-phosphate dehydrogenase. The *in vitro *activity of GAPDH in strain NZ7602 was analyzed and found to be approximately three-fold higher in comparison with the wild-type (Table 2). Furthermore, a gene related to cysteine amino acid metabolism was also upregulated in strain NZ7602 specifically the gene coding for serine O-acetyltransferase (*cysE*).

**Table 3:** Summary of significant affected genes (27) in the *trxB1* over-expression strain, NZ7602, when compared to the wild type (p_value _< 0.01 & FC ≥ 1.5). Predicted gene names, function, fold change induction as well as main functional classes of the significant affected transcripts are displayed in columns. Main functional classes presented in bold are those classes found overrepresented in this study when compared to the total genome of *L. plantarum*.

**Locus**	**FC^2 ^trx/wt**	**Gene**	**Product**	**Main Functional Class (%)^1^**
lp_0254	1,5	*cysE*	serine O-acetyltransferase	**Amino acid biosynthesis (4%)**
lp_3421	1,6		extracellular protein, gamma-D-glutamate-meso-diaminopimelate muropeptidase (putative)	Cell envelope (7%)
lp_2716	1,6	*ica3*	glycosyltransferase (putative)	
lp_0728	0,6	*groEL*	GroEL chaperonin	**Cellular processes (7%)**
lp_2544	1,6	*npr2*	NADH peroxidase	
lp_3020	1,6	*tag2*	DNA-3-methyladenine glycosylase I	**DNA metabolism (4%)**
lp_0789	1,7	*gapB*	glyceraldehyde 3-phosphate dehydrogenase	Energy metabolism (4%)
lp_1237	1,5		unknown	Hypothetical proteins (19%)
lp_1081	1,6		unknown	
lp_1611	2,1		unknown	
lp_1708	2,3		unknown	
lp_1880	3,9		unknown	
lp_1023	1,8		type 4 prepilin-like proteins leader peptide processing enzyme (putative)	**Protein fate (4%)**
lp_2119	1,7	*tuf*	elongation factor Tu	Protein synthesis(4%)
lp_2721	1,5	*purN*	phosphoribosylglycinamide formyltransferase	**Purines, pyrimidines, nucleosides and nucleotides (15%)**
lp_2722	1,6	*purM*	phosphoribosylformylglycinamidine cyclo-ligase	
lp_2729	1,7	*purE*	phosphoribosylaminoimidazole carboxylase, catalytic subunit	
lp_0761	23,5	*trxB1*	thioredoxin reductase (NADPH)	
lp_1230	1,6		transcription regulator	Regulatory function (4%)
lp_3278	1,8		amino acid transport protein	Transport and binding proteins (11%)
lp_1087	1,8		cation transport protein	
lp_2992	2,6	*mntH2*	manganese transport protein	
antilp_3469	1,6			
lp_RNA02	2,6			plasmids (15%)
lp_p2_01	3,1			
lp_p2_02	3,2			
lp_p3_05	1,5			

^1^values given in parenthesis correspond to the percentage of the total amount of genes (27) that belong to each depicted functional class.

^2^FC is fold change or 2^M^

The transcriptome analysis of cells exposed to a hydrogen peroxide pulse allowed us to analyze the effect of hydrogen peroxide on both the *trxB1*-overexpressing and wild-type strains. Oxidative stress significantly affected a total of 267 transcripts with a p_value _< 0.01 and FC ≥1.5 (Additional file 1). We observed that most of the up-regulated transcripts (159) are associated with genes related to defense mechanisms against oxidative challenge: hydrogen peroxide detoxification (*npr2*, *kat*, *trxA2*, *pox3*, *pox5*), exonucleases (*rexA*, *rexB*); stress response (*asp1*, *asp2*, *groES*, *groEL*); DNA repair (*dinP*, *dnaE*, *recA*); putative DNA helicases (lp_0910, lp_0308, lp_0432) and polymerases (*umuC*); transcription regulator repressor of the SOS regulon (*lexA*), ferrous iron transport (*feoA*, *feoB*), as well as an uncharacterized transcription regulator (lp_1360), a manganese transporter (*mntH2*), and the energy metabolism (*gapB*).

Our data showed that hydrogen peroxide stress also resulted in significant down-regulation of the expression of 108 transcripts (Additional file 1). The down-regulated transcripts correspond to genes involved in glucose catabolic pathways: cellular surface proteins; the mannose PTS; energy metabolism (*ndr*, *nar *genes, *adhE*, *fruk*); fatty acid biosynthesis (*fab *genes): acetyl-CoA carboxylases (*accb2*, *accC2*, *accD2*, and *accA2*); nonribosomal peptide bisoynthesis (*nspA*, *nspB*, and *nspC*); sugar uptake (*sacR*, *ccpA*), and 33 prophage genes.

### Regulatory networks

**Figure 4:**
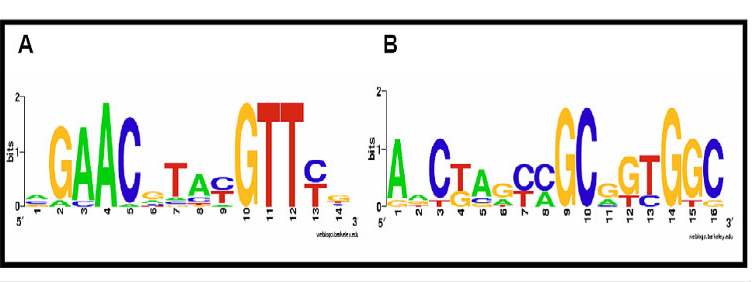
Weblogo representation of conserved promotor regions in peroxide affected genes found using bioinformatics tools A) Regulatory motif *lexA*-*DinR *and B) uncharacterized regulatory motif.

In the group of significantly affected genes due to oxidative stress (Additional file 1) the largest group (22%) corresponded to hypothetical proteins. In order to find a functional correlation in the entire group of peroxide-affected transcripts, we looked for regulatory motifs or binding sites in the upstream region of the affected genes. As a result of this analysis, we found two motifs (Additional file 1) in the upstream region of a number of analyzed sequences. The first motif was found in 15 of the affected genes and had the consensus sequence of the *lexA-DinR *regulator in *B. subtilis *AGAACGTACGTTCG (Fig. 4) [16]. Within the group that contained this promotor sequence, we found well known stress-induced genes (*ruvA*, *ruvB*, *lexA*, *rexA*, *rexB*) [17]. These genes translate into proteins known from literature to be induced under conditions that cause DNA damage or blockage of DNA replication. Also transcripts with hypothetical functions (lp_0091, lp_0270, lp_0030, lp_2224 lp_2939 lp_3141, lp_0981, lp_3022 and lp_1611) contained the *lexA*-*DinR *consensus sequence. It is worth mentioning that these hypothetical transcripts are highly induced under hydrogen peroxide stress. For example, transcript lp_1611 was induced 32-fold as compared to the wild-type.

A second significant regulatory motif denoted here as the Stress Response element (SRE) was found in 7 genes had the consensus sequence AACTAGCCGCGGTGGC (Fig. 4). The important exonucleases: (*rexA*, *rexB*), a DNA polymerase (DNA polymerase III), the transcription regulator *rnhB*, a glycolytic gene (*gapB*), a cell envelope protein, and the hypothetical ORF's lp_0145 and lp_1484 contained this second promotor consensus To the best of our knowledge, this motif has not been reported before.

The same approach was applied to the data set containing the genes affected due to *trxB1*-overexpression. Yet, no significant regulatory motif was found. It can be concluded that genes belonging to the group of hypothetical transcripts (19%) responding to *trxB1*-overexpression do not share a clear single regulatory motif.

### Comparison between oxidative stress response and the effect of *trxB1 *overproduction

Finally, we explored the crossover between the transcriptional response of TR over production with the transcriptional response obtained following hydrogen peroxide treatment of both wild-type and NZ7602. This exercise resulted in a list of 16 transcripts which are affected in both studied conditions (Table 4). The commonly affected transcripts constitute 59% of the transcripts found affected by a trxB1-overexpression (Table 3) and 6% of the genes affected by hydrogen peroxide stress (Additional file 1). From the 16 affected transcripts 94% of them (15) responded similarly to an over production of *trxB1 *as well as to a hydrogen peroxide pulse; these 15 transcripts were up regulated in both datasets. The up-regulated transcripts (15) have already been described and correspond to genes involved in stress related processes (*npr2*), energy metabolism (*gapB*), protein synthesis (*tuf*, *rplB*), manganese uptake (*mntH2*), purine biosynthesis (*trxB1*, and *pur *genes), putative transcript regulators (lp_1360, lp_0126) and unknown transcripts.

**Table 4:** Crossover between genotype affected genes and hydrogen peroxide affected genes. Genes presented in this table are the common transcripts (16) between the two studied datasets: genotype and treatment. Further a summary of the regulation pattern for each of the depicted transcripts is given.

	**FC^1^**					**Transcript Behavior**	
					
**Locus**	**genotype**	**treatment**	**Gene**	**Product**	**Main Functional Class**	**up**	**conflicting**
lp_2716	1,6	0,7	*ica3*	glycosyltransferase (putative)	Cell envelope	•	
lp_0728	0,6	1,7	*groEL*	GroEL chaperonin	Cellular processes		•
lp_2544	1,6	5,0	*npr2*	NADH peroxidase		•	
lp_3020	1,6	0,7	*tag2*	DNA-3-methyladenine glycosylase I	DNA metabolism	•	
lp_0789	1,7	1,8	*gapB*	glyceraldehyde 3-phosphate dehydrogenase	Energy metabolism	•	
lp_1081	1,6	0,5		unknown	Hypothetical proteins	•	
lp_1611	2,1	32,9		unknown		•	
lp_1708	2,3	4,2		unknown		•	
lp_1880	3,9	2,9		unknown		•	
lp_2119	1,7	1,8	*tuf*	elongation factor Tu	Protein synthesis	•	
lp_2721	1,5	3,1	*purN*	phosphoribosylglycinamide formyltransferase	Purines, pyrimidines, nucleosides and nucleotides	•	
lp_2722	1,6	3,4	*purM*	phosphoribosylformylglycinamidine cyclo-ligase		•	
lp_2729	1,7	1,8	*purE*	phosphoribosylaminoimidazole carboxylase, catalytic subunit		•	
lp_0761	23,5	1,8	*trxB1*	thioredoxin reductase (NADPH)		•	
lp_1087	1,8	0,6		cation transport protein	Transport and binding proteins	•	
lp_2992	2,6	1,8	*mntH2*	manganese transport protein		•	

^**1 **^FC is fold change or 2^M^

The only transcript found in both datasets that does not share the same regulation is *groEL*. This transcript is 0.6-fold down regulated as a result of the overexpresssion of *trxB1 *and 1.7-fold upregulated and in response to a hydrogen peroxide pulse.

In order to analyze the effect at the metabolic level, the datasets were superimposed showing they shared two major metabolic pathways: purine metabolism and cysteine biosynthesis.

## Discussion

In this study, we have characterized the role of TR in oxidative stress response of *Lactobacillus plantarum*. We found that overproduction of TR in *L. plantarum *WCFS1 improved the tolerance of the strain towards an oxidative stress produced by hydrogen peroxide or diamide. Global transcriptome analysis revealed a striking similarity in response towards the overproduction of the oxidoreductase TR and hydrogen peroxide stress. Our observations suggest that overproduction of TR triggers the induction of a specific set of 16 transcripts associated with oxidative stress response. This may explain the phenotype of the *trxB1*-overexpression strain, which appears to be prepared for encountering oxidative stress. The transcripts correspond to genes involved in purine metabolism, protein synthesis, as well as in cellular and energy metabolism.

The global transcriptome analysis obtained from cultures affected by hydrogen peroxide stress and in the absence of heme, offers complementary information for the characterization of the *kat*, and *pox *genes in *L. plantarum *WCFS1. The catalase gene (*kat*) of *L. plantarum *WCFS1 is an ortholog of the well characterized manganese-dependent catalase in *L. plantarum *CNRZ 1288 [18]. Accordingly, in our dataset the *kat *transcript and *mntH2*, which codes for a manganese transporter, where both is significantly upregulated three- and two-fold respectively under hydrogen peroxide.

Hence, our transcriptome analysis only supports the fact that the gene catalase (*kat*) of *L. plantarum *WCFS1 may code a pseudocatalase, non-heme dependent catalase, under conditions of hydrogen peroxide stress. Furthermore, under hydrogen peroxide stress the highly upregulated *pox *transcripts (*pox3 *and *pox5*) encoding pyruvate oxidase were at first an intriguing observation. Pyruvate oxidase catalyzes a reaction that utilizes pyruvate, oxygen, phosphate, and water and produces intracellular hydrogen peroxide, carbon dioxide, and acetyl phosphate. Yet, in this study there was no oxygen added because the cultures were grown anaerobically. However, transcripts encoding the genes, catalase (*kat*) and NADPH peroxidase (*npr2*) were also found up regulated. If these two latter enzymes were to be active, they would liberate oxygen and water respectively from hydrogen peroxide and therefore explaining the observed behavior of *pox3 *and *pox5 *under hydrogen peroxide stress.

Hydrogen peroxide not only provokes up-regulation of genes at the transcriptome level. We also observed down-regulation of genes involved in main metabolic pathways: glycolysis, fatty acid biosynthesis, non ribosomal peptide biosynthesis, and amino acid metabolism. This observation suggests that in the presence of hydrogen peroxide stress, the cell responds to it with a reduction in biomass formation. The transcript data suggest that the pathways mentioned above are being kept temporarily "on hold" until the cell has detoxified form the oxidative stress inducing compounds. This reasoning was supported by metabolite analysis data showing interrupted lactate production after exposure to hydrogen peroxide (data not shown).

This study is the first to observe the impact of overproduction of TR at the transcriptome level in *L. plantarum*. The overexpression of *trxB1 *affects the expression level of genes involved in stress related processes, DNA/RNA biosynthesis, and sulfur containing amino acid biosynthesis. The resulting wide spectrum of pathways affected by TR has already been established. Research performed by Vido *et al*. on aerobic growth of *Lactococcus lactis *(2005) revealed that in *L. lactis*, TR is involved not only in oxidative stress but also in carbon and lipid metabolism [19].

Overproduction of TR in *L. plantarum *WCFS1 resulted in regulation of transcripts encoding heat shock and stress related genes. Especially the stress related gene which was found to be down regulated in strain NZ7602 (*groEL*) is previously reported to be induced in *B. subtilis *[20] under stress conditions (heat, salt, and ethanol) together with TRX. In addition, on transcript level *trxA1*, *trxA2*, *trxA3*, and *trxH *were not found significantly affected. This suggests that the protein TRX is not affected at the transcript level in strain NZ7602. Hence, the transcripts regarded as significantly affected in strain NZ7602 are more likely to be a response to the direct manipulation of the TR level and/or the protein load resulting from TR overproduction and not to the change in the TRX balance of the cell.

We have shown that transcript levels of *gapB *and glyceraldehyde-3-phosphate dehydrogenase (GAPDH) activity are significantly increased 1.7-fold and 3-fold, respectively, upon overexpression of *trxB1 *and hydrogen peroxide stress. GAPDH plays an important role and has been considered the key enzyme of glycolysis for its location in the pathway and the number of regulatory interactions associated with this enzyme. A correlation between the GAPDH and TR has already been reported in literature. Vido *et al*., 2005 [21] suggested that a disruption on the *trxB1 *gene in *L. lactis *leads to induction of only one of the two genes that code for the GAPDH protein, specifically *gapB*. Furthermore, the induced GapB was only present in the reduced form. This functionality prevents the formation of oxidized GapB and sustains formation of the reduced or active form of GAPDH. Interestingly, in our case the observed two-fold higher GAPDH activity is a result of overexpression of *trxB1 *in contrast to a disruption in *trxB1 *as reported by Vido *et al *[19] in *L. lactis*. Although overexpression of *gapB *in the strain NZ7602 results in higher reductase activity, the elevated transcript levels under oxidative stress were even more interesting. Studies from Van Niel *et al*., [22] show that the GAPDH protein is an easy target for oxidative stress (cysteine residues) and together with glucokinase, fructose 1–6 biphosphate, and aldolase has been found to be inhibited by 2.2 mM hydrogen peroxide in *L. lactis *[22]. Nevertheless, in our studies, we observed elevated GAPDH activity upon exposure to 3.5 mM hydrogen peroxide. Hence, we can suggest that the functionality of GAPDH is organism dependent. We suggest that overproduction of TR in *L. plantarum *WCFS1 protects the GAPDH under conditions of oxidative stress caused by hydrogen peroxide. However, more studies are needed to understand the role of TR in the regulation of the GAPDH activity.

Using bioinformatics tools we were able to uncover new main stress response ORF's in *L. plantarum *WCFS1. For example the hypothetical ORF lp_1611 which contains the regulatory motif *lexA-dinR*. The ORF lp_1611 was found to be up regulated 32-fold under hydrogen peroxide stress. This ORF is located adjacent to the glycine ABC transporter operon just in the opposite direction of the *opu *genes. The position and direction of lp_1611 may suggest a role as a regulator of transcription; nevertheless, more analysis such as finding the DNA binding region and activity in the protein need to be conducted.

Another group of oxidative-stress-affected genes contains a second regulatory motif that we have termed SRE. In this group we found three genes that had already been defined as *lexA-dinR *regulated genes (*rexA*, *rexB*, *recA*). Moreover, in the group of genes containing the second motif, we found genes involved in primary energy metabolism (*gapB*) and protein synthesis (*rnhb*) implying that there is at least one more regulatory mechanism induced in *L. plantarum *WCFS1 upon oxidative stress. We did not find a unique regulatory motif or SRE motif element in the group of genes affected by overproduction of TR. This reflects the complexity of the regulatory mechanisms associated with TRX or TR. Perhaps, these up-regulated genes are governed by a complex network of signal transduction events which is initiated by the TRX system. Hence, we suggest the set of 16 genes is part of the TR-specific defense mechanism of *L. plantarum *WCFS1 against oxidative stress.

In summary, we have presented evidence that TR is a factor in the oxidative stress response in *L. plantarum *WCFS1. Our transcriptome data suggest that the TRX system (*trxA2 *and *trxB1*) is induced under hydrogen peroxide stress in *L. plantarum *WCFS1. Moreover, the discovery of a crossover-group of genes with a common response under hydrogen peroxide stress, especially DNA-repairing and stress transcripts, including the *pur *genes, *npr2*, and *gapB*, leads to the hypothesis that overproduction of TR results in a "mock-stress-mode." As a result under TR overproduction, 16 transcripts encoding genes involved in purine biosynthesis, cell wall biosynthesis, energy metabolism, cellular envelope biosynthesis, amino acid metabolism are activated. The activation of these genes provides an explanation of why strain *L. plantarum *NZ7602 is better adapted to a challenge with hydrogen peroxide stress in comparison to the wild-type. The observed crossover between TR overproduction and hydrogen peroxide stress contributes essential information to understand the oxidative stress related signal transduction cascade in *L. plantarum*. Nevertheless, this study clearly needs to be followed up with experiments showing the effect, on the group of 16 transcripts resulting from an inactivation of *trxB1 *in *L. plantarum*. New leads concerning catalase (*kat*) and the GAPDH protein emerged from our transcriptome dataset. These leads will be further studied. Using regulatory motif searches we identified a known stress regulatory element: *lexA-dinR *and also a new motif: AACTAGCCGCGGTGGC (Fig. 4). Finally, transcription profiling also revealed new actors such as the uncharacterized ORF lp_1611 which seems to play a role in the oxidative stress response in *L. plantarum *WCFS1. Current investigations aim at pinpointing the role of TR in the oxidative stress response.

## Methods

### Bacterial strains, plasmids, media, and culture conditions

The bacterial strains used in this study are summarized in Table 5. *E. coli *strains were grown at 37°C in TY [23]. *Lactococcus lactis *was grown in M17 at 30°C and *L. plantarum *WCFS1 was grown at 37°C in de Man-Rogosa-Sharp (MRS) or in Chemically Defined Medium (CDM) [24].

**Table 5:** Bacterial strains and plasmids used in this study.

**Strain and Plasmids**	**Characteristics**	**Reference**
**Strains**		
*E. coli *DH5α		
*E. coli *E10		
*L. lactis *NZ9000	MG1363 *pepn::nisRK*	[35, 36]
*L. plantarum *WCFS1	Sequenced wild-type strain single colony isolate of NCIMB8826 form human saliva.	[10]
NZ7100	*L. plantarum *WCFS1 derivative with chromosomal integration of pEMnisRK plasmid.	This work
NZ7606	Cm^R^, *L. plantarum *NZ7100 derivative carrying the pNZ8150 plasmid.	This work
NZ7601	Cm^R^, *L. plantarum *NZ7100 derivative carrying the pMS011 plasmid.	This work
NZ7607	Cm^R^, *L. plantarum *WCFS1 derivative carrying the pNZ7021 plasmid.	This work
NZ7602	Cm^R^, *L. plantarum *WCFS1 derivative carrying the pMS040 plasmid.	This work
**Plasmids**		
pUC18Ery	Amp^R^, Em^R^	[26]
pACYC184	Cm^R^, Tet^R^	[37]
pNZ84	Cm^R^, pACYC184 derivative with deletion of Tetracycline resistance gene and BamhI site.	[38]
pNZ9521	Em^R^, nisRK cloned in pIL253, expression of nisRK driven by rep read-through.	[27]
pNZ7130	Amp^R^, Em^R ^pUC18EM derivative carrying 1.0 kb DNA fragments of both *L. plantarum *WCFS1 lp_0075 and lp_0077 genes.	This work
pNZ7131	Amp^R^, Em^R^, pNZ7130 derivative with extra cloning sites Nsa1 and NsiI.	This work
ppNZ7132	Cm^R^, Em ^R^, pNZ84 derivative carrying 1.0 kb DNA fragments of *L. plantarum *WCFS1 (lp_0075) and (lp_0077) genes and the Ery^R ^resistance marker.	This work
pNZ7133	Cm^R^, Em ^R ^p7132 derivative carrying the whole genes nisRK from L. lactis.	This work
pNZ8150	Cm^R^, pNZ8148 derivative lactococcal cloning and expression vector with nisA promoter upstream of a multiple cloning site and Sca1 restriction site.	[39]
pMS011	Cm^R^, pNZ8150 derivative carrying *L. plantarum *(lp_0761) *trxB1 *gene, translation fused to the nisA promoter.	This work
pNZ7021	Cm^R^, pNZ8148 derivative carrying *nisA::pepN*.	[40]
pMS040	Cm^R^, pNZ7021 derivative carrying *L. plantarum *lp_0761 *trxB1 *gene, translational fused to the pP_EPN _promoter.	This work

### DNA Manipulations

All molecular biology techniques were performed following established protocols by Sambrook [23]. DNA was digested according to the conditions recommended by the commercial suppliers of the restriction enzymes (Boehringer, Breda, The Netherlands). In all cases, DNA was eluted from 0.7% agarose gels using the Purification Kits from Promega (Leiden, The Netherlands). For a Polymerase chain reaction (PCR) we used: 1 μl template DNA (10 to 100 ng), 2 μl of each primer combination (50 ng/ml), 1 μl dNTP's (100 nM), 1 μl Pwo-polymerase (5 U/μl) and 10 μl polymerase buffer (5×) (Roche, Woerden, The Netherlands). The reaction mixtures were adjusted to 50 μl with deionized H_2_O.

### Construction of strain L. plantarum NZ7100

Previously it has been established that the nisin controlled expression system can be functionally implemented in *L. plantarum*, by chromosomal integration of the regulatory module encoding genes *nisRK *[25]. However, the strain described by Pavan *et al*. harbors an erythromycin resistance marker. Therefore, a chromosomal *lp_0076:::nisRK *gene replacement without resistance marker was constructed. For this purpose, lp_0076 upstream and downstream flanking regions were amplified by proofreading PCR using genomic DNA of *L. plantarum *WCFS1 as template. The primers used to amplify the 5'- and 3'-regions of lp_0075 and lp_0077 respectively were lp_0075-FORW and lp_0075-REV; lp_0077-FORW and lp_0077-REV, shown in Table 6. The PCR amplicons obtained were digested with EcorI-BamhI, and BamhI-XbaI (sites introduced in the primers used are underlined in Table 6) and cloned into a EcoRI-XbaI digested pUC18Em [26]. The genetic organization of the resulting plasmid was verified by PCR and was designated pNZ7130. To introduce additional endonuclease restriction sites in pNZ7130 the multiple cloning sites containing fragment was amplified by PCR using the primers EM1 and EM2 (Table 6). The resulting amplicon (35 bp) was digested with BamhI and BglII and cloned in BamhI digested pNZ7130, yielding pNZ7131. The 2.6 kb HindII fragment of pNZ7131 that contains the lp_0076 flanking regions separated by the multiple cloning site and the erythromycin resistance encoding gene, was subcloned in vector pNZ84, yielding the lp_0076 replacement vector pNZ7132. The *lp_0076::nisRK *replacement plasmid pNZ7133 was constructed by cloning of the 2.4 kb HpaII-PstI fragment of pNZ9521 [27] into the NsaI-NsiI digested pNZ7132. The plasmid pNZ7133 was introduced into *L. plantarum *WCFS1 and primary integrants were selected on basis of erythromycin resistance. The anticipated configuration of the pNZ7133 plasmid integration in the lp_0076 locus was confirmed by PCR and Southern blotting (data not shown). One of the integrants was cultured for 140 generations without antibiotic selection. Subsequently, erythromycin sensitive (Em^S^) colonies were identified by plating on media without erythromycin followed by replication plating on erythromycin containing plates. In the Em^S ^colonies identified, a candidate *lp_0076::nisRK *replacement mutant was identified by PCR using internal primers in *NisK *and *Orfx *(data not shown). In a selected candidate *lp_0076::nisRK *strain, the anticipated genetic organization of the *lp_0076::nisRK *locus was further confirmed by additional PCR and Southern blot analyses (data not shown). The *lp_0076::nisRK *derivative of *L. plantarum *WCFS1 was designated NZ7100.

**Table 6:** Oligonucleotides used in this study. Designed restriction sites in the primers are shown underlined in the table.

**Oligonucleotides (5'-3')**	**Sequence**	**References**
Lp_0075-FORW	ACGTGAATTCCAGTTCAACTAGAACAAGC	
Lp_0075-REV	ACGTGGATCCTACAATCCGTTTCATATTG	
Lp_0077-FORW	ACGTGGATCCAAGGCGTCATCAAAATAG	
Lp-0077-REV	ACGTTCTAGACGCAATTTTCTTCACATTAC	
EM1	GGATCCGTTAACGAATTCGGCGCGCCGGCGCCA	
EM2	AGATCTGGCGCCGGCGCGGCGAATTCGTTAACG	
*trxB1*-FORW	ATGGCAAAGAGTTACGACG	
*trxB1*-REV	GTTGCATGCTCAATTAA ATCAGCTACGC	
*trxB1*-300	TTC AGA ACC AGT CCC AAT GAC	
*trxB1*-KPN1FORW	CGCCCACGGTACCATGGCAAAGAGTTACGACG	
*trxB1*-XBA1REV	CGCGCCTCTAGACTCAATTAAATCAGCTACGC	
*trxA1*-FORW	ATGATCGAACCAGTCGATAAG	
*trxA1*-REV	TTAGGCAGGCTTC ACTTC	
*trxA2*-FORW	ATGGTCGCAGCAACTACTG	
*trxA2*-REV	TTATAGATA TTGAGCTAAAGTTTG	
*trxA3*-FORW	ATGATTAAAGAAATACATGACC	
*trxA3*-REV	TTAAGCAAGTGCTTTTTTGAGTTC	
*trxH*-FORW	ATGGCAACTGCA ACTTTAAG	
*trxH*-REV	CTACTTTTCAAGTGTATCTAAG	
*trxB2*-FORW	ATGAGTGCAGAATATGATTTAAC	
*trxB2*-REV	CGGGTACCCCGGTTTCTCGTCCTATTTGC	
*trxB2*-truncFORW	ATT CGG ATC CGT AGT CTC TGC AAG TTG C	
*trxB2*-truncREV	TTT GCG GTA CCA CAG AAT ATG ATT TAA CAA TTA	

### Construction of trxB1 over-expressing strains NZ7601 and NZ7602

#### NZ7601

The gene *trxB1 *in *L. plantarum *WCFS1 was amplified by Pwo-polymerase using genomic DNA from *L. plantarum *WCFS1 as template and two primers: *trxB1*-FORW and *trxB1*-REV with a SphI restriction site (site introduced in the primer used; underlined Table 6). The amplified DNA fragment was cloned into the linearized vector pNZ8150. The complete plasmid, designated pMS011, was cloned and purified from *L. lactis *NZ900 cells [28] displaying chloramphenicol resistance (Cm^R^) and introduced into *L. plantarum *NZ7100. The plasmid isolated form the *L. plantarum *colonies displaying Cm^R ^were checked by restriction and sequencing analysis. One of these transformants containing the *L. plantarum trxB1 *gene translationally fused to the *nisA *promoter was denominated NZ7601.

#### NZ7602

The gene *trxB1 *in *L. plantarum *WCFS1 was amplified by Pwo-polymerase using genomic DNA from *L. plantarum *WCFS1 as template and two primers: *trxB1*-KPNFORW and *trxB1*-XBA1REV containing a Kpn1 and Xba1 site, respectively. The amplified fragment was purified from the gel and cloned into the digested vector pNZ7021. The complete plasmid, pMS040, was cloned and isolated from *L. lactis *NZ9000 colonies displaying Cm^R ^From the colonies displaying Cm^R ^plasmid was isolated and checked by restriction and PCR analysis. Then, purified plasmid pMS040 was inserted into *L. plantarum *NZ7100 [29]. These transformants contained the *trxB1 *gene translationally fused with the constitutive P_pepN _promoter [15] and one of this colonies was denominated NZ7602.

### Control strains NZ7606 and NZ7607

In the experiments with the nisin inducible promoter we used as control strain NZ7606, a *L. plantarum *NZ1700 strain containing the pNZ8150 vector. On the other hand, for the chemostat studies strain NZ7607 was used as control. Strain NZ7607 is *L. plantarum *WCFS1 containing the pNZ7021 vector.

### Nisin Induction

Nisin induction was done as previously described by Pavan *et al *[25] using 50 ng/ml nisin for induction.

### Quantitative PCR assays

Total RNA was isolated from exponentially growing *L. plantarum *WCFS1 cultures with the High Pure RNA Isolation Kit (Roche, Woerden, The Netherlands). To eliminate genomic DNA contamination a 60 min DNAse I treatment was included in the isolation procedure. Furthermore, the quality and quantity of the RNA was confirmed using the RNA 6000 Nano Assay (Agilent Technologies, Amstelveen, The Netherlands) using the Agilent 2100 expert Bioanalyzer. Cultures of the bacterium were grown in anaerobic jars on CDM containing 5 mM Diamide at 37°C, or CDM containing 5 mM DTT at 37°C and 30°C. In all cases 0.5% (w/v) glucose was added as carbon source. For the reverse transcription reaction 200 ng RNA was incubated at 65°C for 5 minutes with 270 ng random nonamers, and 1 μl of 10 mM dNTP mixture. After 5 minutes on ice, the following was added: 5 μl 5 × First strand Buffer, 2 μl 0.1 M DTT, 1 μl Superscript III (200 UNITS), 1 μl RNASEout (40 UNITS) (all Invitrogen) and water to a final volume of 20 μl. The reaction was incubated at 25°C for 5 min and then at 50°C for 60 min. The reaction was inactivated by heating at 70°C for 15 min. Generated cDNA samples were stored at -20°C until use. Quantitative PCR amplification was performed in 96-well plate on a 7500 Fast System (Applied Biosystems), using SYBR green for product detection. Each well contained 10 μl SYBR green Master Mix (Applied Biosystems), 200 nM of Reverse and forward primers, and 1 μl of 10-fold or 100-fold diluted RT product as template. The qPCR gene-specific probes of *L. plantarum *WCFS1 of *trxA1 *(0.3 kb), *trxA2 *(0.3 kb), *trxA3 *(0.3 kb), *trxH *(0.3 kb), *trxB1 *(0.3 kb), and *trxB2 *(0.6 kb) were amplified using specific primer combinations *trxA1*-FORW and *trxA1*-REV; *trxA2*-FORW and *trxA2*-REV; *trxA3*-FORW and trxA3-REV; *trxH*-FORW and *trxH*-REV; *trxB1*-FORW and *trxB1*-300; *trxB2*-truncFORW and *trxB2*-truncREV. All primers are specified in Table 6. Amplification was initiated at 95°C for 10 min, followed by 40 cycles of 95°C for 15 sec, 53°C for 30 sec and 60°C for 60 sec for probes *trxA1*, *trxA3 *and *trxH*. For the other probes (*trxA2*, *trxB1*, and *trxB2*) the step of 53°C for 30 sec was changed to 55°C for 30 sec. All samples were measured in duplicate. Control PCRs were included to detect background contamination (no-template control) and remaining chromosomal DNA (RT reactions in which enzyme superscript III was not added). PCR specificity and product detection were checked post amplification by examining the dissociation curves of the PCR products. These melting curve profiles were generated by first heating the samples to 95°C and then cooling them to 60°C and slowly heating then at 2°C/min to 95°C for detection of SYBR green fluorescence. In each run, five standards of the gene of interest were included with appropriate dilutions of the cDNA, to determine the cDNA concentration in the samples. All q-PCRs amplified a single product as determined by the melting curve analysis and the releative expression level is given as arbitrary units (Au).

Growth curves. For the growth experiments, 96-well plates were used. Each well was filled with 200 μl medium and 10 μl growing cells (OD_600 _of 1.0). The 96-well plates were inoculated at 37°C and cell density was measured by detecting the turbidity of the cultures at 600 nm every 10 min. To study oxidative stress response, either diamide (5 mM) or hydrogen peroxide (3.5 mM) was added to the medium.

### Growth-zone inhibition assays

Cultures were grown at 37°C in MRS until OD_600 _of 0.4. At this point, 2.5 ml of culture was plated by mixing with 50 ml of 0.7% Agarose MRS at 40°C. After the agar/culture plates hardened, perforations of 6 mm diameter were made. In each of these apertures, 30 μl of solution (1 M, 0.5 M, 0.25 M and 0.10 M) of diamide or (1 M, 0.5 M) hydrogen peroxide was added. Plates were incubated overnight at 37°C. The growth inhibition towards the oxidative stress agents was measured as the radius of growth inhibition expressed in centimeters.

### Preparation of cell free extracts (CFE)

Cells of a growing culture OD_600 _of 1.0 (approx. 10 ml) were harvested by centrifugation at 12.000 × g for 10 minutes at 4°C. The pellet was washed twice with 1 M Tris-HCl buffer pH = 7.5. Next, the cells were suspended in 1 ml of 1 M Tris-HCl buffer pH = 7.5 and disrupted by Fastprep (Qbiogene Inc., Illkirch, France) in four treatments of 40 seconds at speed 4.0. The suspension was centrifuged 2 min at maximum speed and the supernatant or CFE was transferred to a new vial and used directly. Protein content of the CFE was determined through the standard BCA Protein Assay kit (Pierce, Etten-Leur, The Netherlands).

### Thioredoxin reductase enzyme assays

The reaction mixture (RM) was prepared by mixing (in ice) 200 μl of 1 M Tris-HCl, pH = 7.5, 500 μl of insulin (10 μg/μl), 2.5 μl EDTA pH = 7.5 (0.2M), and 40 μl NADPH (40 mg/ml). For each enzymatic reaction 40 μl RM were used together with 20 μl TRX (60 μM) from *E. coli *(Sigma), and 80 μl CFE. Each reaction was incubated for 25 minutes at 37°C; after this 500 μl of 6M Guanidine HCl/0.2 M Tris-HCl, pH = 7.5/1 mM DTNB was added. The final absorbance at 412 nm was measured at 25°C. Note that the extinction coefficient of DTNB reduction is 13.6 mM^-1 ^and that there are two molecules of TNB per molecule DTNB being reduded. The background signal in the assay was determined in the absence of TRX in one of the assays. TR activity was expressed as nmol of DTNB·(min·mg protein)^-1 ^and calculated as follows: Δabs_412_·(0.620)·(protein content)^-1^·2·(εDTNB)^-1^·10^^6^.

### Glyceraldehyde-3-phosphate dehydrogenase enzyme assay

The assay mixture contained: triethanolamine-HCl buffer (pH 7.6), 100 mM; ATP, 1 mM; EDTA, 1 mM; MgSO4, 1.5 mM; NADH, 0.15 mM; phosphoglycerate kinase 22.5 U (Boehringer, Breda, The Netherlands); and CFE. The reaction was started with 5 mM 3-phosphoglycerate. The absorption of the assay mixture was monitor at 340 nm (E_340 _nm of reduced pyridine-dinucleotide cofactors is 6.3 mM^-1^). Enzyme activity is expressed as μM of amount of NADH converted per (min·mg protein)^-1^. All assays were performed with two concentrations of cell extract to confirm that reaction rates were proportional to the amount of cell extract added. Protein content of the CFE was determined through the standard BCA Protein Assay kit.

### Continuous cultivations

Cultures were grown at 37°C in CDM [24] which was supplemented with 100 mM glucose and 10 μg/ml of chloramphenicol. A 1 Liter bioreactor (Applikon Dependable Instruments) was inoculated with cells from an overnight culture to an initial OD_600 _of 0.1 in 500 ml medium. A pH of 5.5 was maintained by the addition of 5 M NaOH and the stirrer speed was set at 200 rotations per minute (rpm). The headspace of the fermentors and medium vessel were full at all times with nitrogen gas at a flow rate of 520 ml·min^-1^. The cultures were kept at the dilution rate of 0.1 h^-1 ^and steady state was assumed after 5 volume changes. At steady state or (t_0_), samples were taken for transcriptomics, HPLC analysis, dry weight, and enzyme samples. In addition, at steady state, 50 ml of hydrogen peroxide was added to the fermentors resulting in a final concentration of 3.5 mM in the fermentor. After 30 min (t_30_), samples were taken for transcriptomics, HPCL analysis, and enzymatic analysis.

### Microarrays

#### RNA isolation

A 40 ml *L. plantarum *WCFS1 culture at steady state was added to 160 ml of quenching buffer [30] (60% methanol, 66.7 mM HEPES; pH 6.5; 40°C). Following quenching, the cells were immediately pelleted by centrifugation at 18.000 × g for 10 min at 20°C. The pellet was resuspended in a screw cap tube containing in 0.4 ml TE, 250 μl acidic phenol, 250 μl chloroform, 30 μl 3 M sodium acetate pH 5.2, 30 μl 10% sodium dodecyl sulphate, and 1 gram glass beads. The tube was immediately frozen in liquid nitrogen and samples were stored at -80°C. The cells were disrupted with three subsequent 40 second treatments separated by 1 min on ice in a Fastprep (Qbiogene Inc., Illkirch, France). After disruption, 0.5 ml of the aqueous phase was used for RNA isolation with a High Pure kit, which included 1 h of treatment with DNase I (Roche Diagnostics, Mannheim; Germany). The isolated RNA was eluted in 50 μl of elution supplied in the kit.

#### cDNA synthesis and purification

Before first-strand cDNA synthesis, the absence of genomic DNA and RNA degradation in the RNA samples was confirmed using the RNA 6000 Nano Assay (Agilent Technologies, Amstelveen, The Netherlands) using the Agilent 2100 expert Bioanalyzer. First-strand cDNA synthesis was carried out by the CyScribe Post-Labelling and Purification kit (Amersham Biosciences, Buckinghamshire, UK) following the manufacturer's instructions with two modifications. The starting amount of mRNA used per sample was 25 μg and the synthesis reactions were incubated for 3 h at 42°C.

#### Data acquisition and processing

Two independent chemostat cultivations were performed for both wild type and *trxB1 *over-expression strain. Three hybridization experiments, all with the same hybridization scheme (see below), were performed with samples obtained from these chemostats before and after treatment with hydrogen peroxide. Two of these experiments used samples obtained from the same fermentation. In each experiment three arrays were used. Per array two cDNA labeled targets were hybridized on custom designed *L. plantarum *WCFS1 11 K Agilent oligo microarrays (GEO Acc. Nr. GPL4318) using the Agilent 60-mer oligo microarray processing protocol version 4.1. These microarrays contained an average of three probes per gene. The hybridization scheme contained the following cDNA comparisons (1) wildtype with *trxB1 *over-expression mutant; (2) wildtype with wildtype treated with hydrogen peroxide; and (3) wildtype treated with hydrogen peroxide with *trxB1 *over-expression mutant treated with hydrogen peroxide. Dried slides were scanned in the Scan Array Express (PerkinElmer Life Sciences; Packard Bioscience) at 10 microns. Spot intensity data was quantified (average intensity) in ImaGene version 5.0 (BioDiscovery, Inc., El Segundo, CA). Signal intensities of all probes were corrected against background and normalized by fitting a plot of M (= _2_log [cy5 intensity/cy3 intensity]) against A (= 0.5·_2_log [cy5 intensity·cy3 intensity]) using the lowess algorithm in BASE [31]. The normalized data has been made available with GEO Acc. Nr. GSE8348. The fold change (FC) is defined as 2^M^. For the statistical analysis we used microarray analysis of variation (R/maanova) [32]. In this maanova test we used three variables: fermentation, treatment, and genotype. Moreover we tested the model taking into consideration the interaction between genotype and treatment. The maanova test resulted only in two sets of interesting data because the interaction effect did not reveal significant changes in transcript levels. One dataset representing the transcripts affected as a result of the overproduction of TR and another set representing the transcripts affected due to oxidative stress. These two datasets were denominated genotype and hydrogen peroxide datasets respectively. Significantly regulated genes within each dataset were defined as genes whose nominal p_values _were less than 1% and had a fold change equal or higher than 1.5.

### Regulatory Motifs

Determination of regulatory motifs from a list of genes was based on predictions of upstream transcriptional units (TU's) made using an in-house Python program, alignments of these TU's using the MEME [33] and determining of nucleotide sequences using the MAST software [34] tools. The MEME software determines through alignments small motifs which are present in the analyzed sequences and assigns a significant value, E_value_, and score per position. This significant value represents how well preserved the motif is, the number of TU's that this motif has, and the conserved position of the motif in the analyzed TU's. The MAST software allows searching for the motif in the genome of interest.

## Competing interests

The authors(s) declare that they have no competing interests.

## Authors' contributions

The presented experimental work was performed by LMS, a PhD scholar working under the supervision of ES and WdV. PB constructed strain NZ7100. DM performed the statistical analysis of the microarrays. Regulatory Motif search was done by MW. For the visualization of transcriptome data we used the genome scale model of *L. plantarum *WCFS developed by BT. All authors read and approved the final manuscript.

## Supplementary Material

Additional file 1 Global transcriptome response towards hydrogen peroxide stress in *L. plantarum *strains NZ7607 and NZ7602. Significantly affected genes (267) due to hydrogen peroxide stress both in strains NZ7607 and NZ7602 (p_value _< 0.01 & FC ≥ 1.5). Predicted gene names, function, fold change induction as well as main class of the genes are displayed in column one and three respectively. Main functional classes presented in bold are those classes found overrepresented in this study when compared to the total genome of *L. plantarum*. Click here for file

## References

[B1] Laurent TC, Moore EC, Reichard P (1964). Enzymatic Synthesis of Deoxyribonucleotides. Iv. Isolation and Characterization of Thioredoxin, the Hydrogen Donor from Escherichia Coli B. J Biol Chem.

[B2] Arner ES, Holmgren A (2000). Physiological functions of thioredoxin and thioredoxin reductase. Eur J Biochem.

[B3] Prieto-Alamo MJ, Jurado J, Gallardo-Madueno R, Monje-Casas F, Holmgren A, Pueyo C (2000). Transcriptional regulation of glutaredoxin and thioredoxin pathways and related enzymes in response to oxidative stress. J Biol Chem.

[B4] Stoyanovsky DA, Tyurina YY, Tyurin VA, Anand D, Mandavia DN, Gius D, Ivanova J, Pitt B, Billiar TR, Kagan VE (2005). Thioredoxin and lipoic acid catalyze the denitrosation of low molecular weight and protein S-nitrosothiols. J Am Chem Soc.

[B5] Jobin MP, Garmyn D, Divies C, Guzzo J (1999). Expression of the Oenococcus oeni trxA gene is induced by hydrogen peroxide and heat shock. Microbiology.

[B6] Scharf C, Riethdorf S, Ernst H, Engelmann S, Volker U, Hecker M (1998). Thioredoxin is an essential protein induced by multiple stresses in Bacillus subtilis. J Bacteriol.

[B7] Vignols F, Brehelin C, Surdin-Kerjan Y, Thomas D, Meyer Y (2005). A yeast two-hybrid knockout strain to explore thioredoxin-interacting proteins in vivo. Proc Natl Acad Sci USA.

[B8] Holmgren A (1985). Thioredoxin. Annu Rev Biochem.

[B9] Li Y, Hugenholtz J, Sybesma W, Abee T, Molenaar D (2005). Using Lactococcus lactis for glutathione overproduction. Appl Microbiol Biotechnol.

[B10] Kleerebezem M, Boekhorst J, van Kranenburg R, Molenaar D, Kuipers OP, Leer R, Tarchini R, Peters SA, Sandbrink HM, Fiers MW (2003). Complete genome sequence of Lactobacillus plantarum WCFS1. Proc Natl Acad Sci USA.

[B11] Molenaar D, Bringel F, Schuren FH, de Vos WM, Siezen RJ, Kleerebezem M (2005). Exploring Lactobacillus plantarum genome diversity by using microarrays. J Bacteriol.

[B12] Seo D, Kamino K, Inoue K, Sakurai H (2004). Purification and characterization of ferredoxin-NADP+ reductase encoded by Bacillus subtilis yumC. Arch Microbiol.

[B13] de Ruyter PG, Kuipers OP, de Vos WM (1996). Controlled gene expression systems for Lactococcus lactis with the food-grade inducer nisin. Appl Environ Microbiol.

[B14] Leichert LI, Scharf C, Hecker M (2003). Global characterization of disulfide stress in Bacillus subtilis. J Bacteriol.

[B15] Tan PS, van Alen-Boerrigter IJ, Poolman B, Siezen RJ, de Vos WM, Konings WN (1992). Characterization of the Lactococcus lactis pepN gene encoding an aminopeptidase homologous to mammalian aminopeptidase N. FEBS Lett.

[B16] Winterling KW, Chafin D, Hayes JJ, Sun J, Levine AS, Yasbin RE, Woodgate R (1998). The Bacillus subtilis DinR binding site: redefinition of the consensus sequence. J Bacteriol.

[B17] Winterling KW, Levine AS, Yasbin RE, Woodgate R (1997). Characterization of DinR, the Bacillus subtilis SOS repressor. J Bacteriol.

[B18] Abriouel H, Herrmann A, Starke J, Yousif NM, Wijaya A, Tauscher B, Holzapfel W, Franz CM (2004). Cloning and heterologous expression of hematin-dependent catalase produced by Lactobacillus plantarum CNRZ 1228. Appl Environ Microbiol.

[B19] Vido K, Diemer H, Dorsselaer AV, Leize E, Juilard V, Gruss A, Gaudu P (2005). Roles of Thioredoxin Reductase during the Aerobic Life of Lactococcus lactis. Journal of Bacteriology.

[B20] Scharf C, Riethdorf S, Ernst H, Engelmann S, Volker U, Hecker M (1998). Thioredoxin is an essential protein induced by multiple stresses in Bacillus subtilis. J Bacteriol.

[B21] Vido K, Diemer H, Van Dorsselaer A, Leize E, Juillard V, Gruss A, Gaudu P (2005). Roles of thioredoxin reductase during the aerobic life of Lactococcus lactis. J Bacteriol.

[B22] van Niel EW, Hofvendahl K, Hahn-Hagerdal B (2002). Formation and conversion of oxygen metabolites by Lactococcus lactis subsp. lactis ATCC 19435 under different growth conditions. Appl Environ Microbiol.

[B23] Sambrook JF, EF, Maniatis T (1989). Molecular Cloning: A Laboratory manual.

[B24] Teusink B, van Enckevort FH, Francke C, Wiersma A, Wegkamp A, Smid EJ, Siezen RJ (2005). In silico reconstruction of the metabolic pathways of Lactobacillus plantarum: comparing predictions of nutrient requirements with those from growth experiments. Appl Environ Microbiol.

[B25] Pavan S, Hols P, Delcour J, Geoffroy MC, Grangette C, Kleerebezem M, Mercenier A (2000). Adaptation of the nisin-controlled expression system in Lactobacillus plantarum: a tool to study in vivo biological effects. Appl Environ Microbiol.

[B26] van Kranenburg R, Marugg JD, van S, Willem NJ, de Vos WM (1997). Molecular characterization of the plasmid-encoded eps gene cluster essential for exopolysaccharide biosynthesis in Lactococcus lactis. Mol Microbiol.

[B27] Kleerebezem M, Beerthuyzen MM, Vaughan EE, de Vos WM, Kuipers OP (1997). Controlled gene expression systems for lactic acid bacteria: transferable nisin-inducible expression cassettes for Lactococcus, Leuconostoc, and Lactobacillus spp. Appl Environ Microbiol.

[B28] Holo H, Nes IF (1989). High-Frequency Transformation, by Electroporation, of Lactococcus lactis subsp. cremoris Grown with Glycine in Osmotically Stabilized Media. Appl Environ Microbiol.

[B29] Josson K, Scheirlinck T, Michiels F, Platteeuw C, Stanssens P, Joos H, Dhaese P, Zabeau M, Mahillon J (1989). Characterization of a gram-positive broad-host-range plasmid isolated from Lactobacillus hilgardii. Plasmid.

[B30] Pieterse B, Jellema RH, van der Werf MJ (2006). Quenching of microbial samples for increased reliability of microarray data. J Microbiol Methods.

[B31] Saal LHTC, Vallon-Christersson J, Gruvberger S, Borg A, Peterson C (2002). "BioArray Software Environment (BASE): a platform for comprehensive management and analysis of microarray data.". Genome Biol.

[B32] Cui X, Churchill GA (2003). Statistical tests for differential expression in cDNA microarray experiments. Genome Biol.

[B33] Elkan TLBaC (1994). "Fitting a mixture model by expectation maximization to discover motifs in bioploymers". Proceedings of the Second International Conference on Intelligent Systems for Molecular Biology.

[B34] Gribskov TLBaM (1998). "Combining evidence using p-values:application to sequence homology searches". Bioinformatics.

[B35] Bolotin A, Wincker P, Mauger S, Jaillon O, Malarme K, Weissenbach J, Ehrlich SD, Sorokin A (2001). The complete genome sequence of the lactic acid bacterium Lactococcus lactis ssp. lactis IL1403. Genome Res.

[B36] Gasson MJ (1983). Plasmid complements of Streptococcus lactis NCDO 712 and other lactic streptococci after protoplast-induced curing. J Bacteriol.

[B37] Chang AC, Cohen SN (1978). Construction and characterization of amplifiable multicopy DNA cloning vehicles derived from the P15A cryptic miniplasmid. J Bacteriol.

[B38] van Alen-Boerrigter IJ, Baankreis R, de Vos WM (1991). Characterization and overexpression of the Lactococcus lactis pepN gene and localization of its product, aminopeptidase N. Appl Environ Microbiol.

[B39] Mierau I, Kleerebezem M (2005). 10 years of the nisin-controlled gene expression system (NICE) in Lactococcus lactis. Appl Microbiol Biotechnol.

[B40] Wegkamp A, van Oorschot W, de Vos WM, Smid EJ (2007). Characterization of the role of para-aminobenzoic acid biosynthesis in folate production by Lactococcus lactis. Appl Environ Microbiol.

