# Supplementary material for: Thioredoxin reductase is a key factor in the oxidative stress response of Lactobacillus plantarum WCFS1
Source: Microb Cell Fact. 2007 Aug 28;6:29. doi: 10.1186/1475-2859-6-29 (PMC2174512; doi:10.1186/1475-2859-6-29)
Supplement: Additional file 1 — Global transcriptome response towards hydrogen peroxide stress in L. plantarum strains NZ7607 and NZ7602. Significantly affected genes (267) due to hydrogen peroxide stress both in strains NZ7607 and NZ7602 (pvalue < 0.01 & FC ≥ 1.5). Predicted gene names, function, fold change induction as well as main class of the genes are displayed in column one and three respectively. Main functional classes presented in bold are those classes found overrepresented in this study when compared to the total genome of L. plantarum. [file 1475-2859-6-29-S1.pdf]

Locus	FC[2] treatment	Gene	Product	Main Functional Class (%)[1]
lp_0979	1,52	*thrA1*	aspartate kinase	Amino acid biosynthesis (1%)
lp_2034	1,54	*tyrA*	prephenate dehydrogenase	
lp_0255	1,86	*metC1*	cystathionine beta-lyase	
lp_0256	1,99	*cysK*	cysteine synthase	
lp_1492	0,66	*moaC*	molybdopterin precursor synthase MoaC	Biosynthesis of cofactors, prosthetic groups, and carriers (1%)
lp_2377	1,51	*hemK*	protoporphyrinogen oxidase (putative)	
lp_1470	1,95	*csd1*	cysteine desulfurase	
lp_2270	2,06	*trxA2*	thioredoxin	
lp_3154	0,52	*acm3-C*	muramidase, C-terminal fragment	Cell envelope (4%)
lp_3679	0,57		extracellular protein	
lp_3676	0,62		extracellular protein	
lp_3677	0,63		cell surface protein precursor	
lp_1165	0,65		cell surface hydrolase (putative)	
lp_0197	0,65		cell surface protein precursor	
lp_2716	0,66	*ica3*	glycosyltransferase (putative)	
lp_0304	1,62		extracellular protein	
lp_2658	1,69		glycosyltransferase (putative)	
lp_1793[3]	1,89		adherence protein	
lp_0774	1,57	*luxS*	autoinducer production protein	Cellular processes (3%)
lp_0728	1,66	*groEL*	GroEL chaperonin	
lp_3128	1,85		stress induced DNA binding protein	
lp_0727	1,93	*groES*	GroES co-chaperonin	
lp_0930	2,28	*asp2*	alkaline shock protein	
lp_0929	2,47	*asp1*	alkaline shock protein	
lp_3578	3,25	*kat*	catalase	
lp_2906	3,68	*endA*	DNA-entry nuclease	
lp_2544	5,01	*npr2*	NADH peroxidase	
lp_2610	1,51	*dxs*	1-deoxy-D-xylulose-5-phosphate synthase	Central intermediary metabolism (1%)
lp_3263	1,53	*crtM*	phytoene synthase	
lp_3020	0,66	*tag2*	DNA-3-methyladenine glycosylase I	**DNA metabolism (7%)**
lp_2109	1,52	*uvrC*	excinuclease ABC, subunit C	
lp_0910	1,57		DNA helicase (putative)	
lp_1885	1,62	*recQ2*	ATP-dependent DNA helicase RecQ	
lp_0811	1,69		DNA-directed DNA polymerase III, epsilon chain (putative)	
lp_2286[2]	1,77	*ruvB*	holliday junction DNA helicase RuvB	
lp_2287[2]	1,78	*ruvA*	holliday junction DNA helicase RuvA	
lp_1840	1,86	*parE*	topoisomerase IV, subunit B	
lp_1899	1,93	*dnaE*	DNA-directed DNA polymerase III, alpha chain	
lp_0432	2,12		DNA helicase (putative)	
lp_0308	2,14		DNA helicase (putative)	
lp_0773	2,21	*uvrA1*	excinuclease ABC, subunit A	
lp_1839	2,35	*parC*	topoisomerase IV, subunit A	
lp_2693[2,3]	2,60	*rexA*	ATP-dependent nuclease, subunit A	
lp_2694[2,3]	2,69	*rexB*	ATP-dependent nuclease, subunit B	
lp_0772	2,77	*uvrB*	excinuclease ABC, subunit B	
lp_2301[2,3]	4,11	*recA*	recombinase A	
lp_2280	4,35	*dinP*	DNA-damage-inducible protein P	
lp_3023	4,62	*umuC*	UV-damage repair protein	

Locus	FC[2] treatment	Gene	Product	Main Functional Class (%)[1]
lp_3662	0,45	*adhE*	bifunctional protein: alcohol dehydrogenase; acetaldehyde dehydrogenase	
lp_0579	0,45	*panD*	aspartate 1-decarboxylase	
lp_2684	0,50	*araT2*	aromatic amino acid specific aminotransferase	
lp_1912	0,55	*pps*	pyruvate,water dikinase	
lp_3605	0,56	*iolG1*	myo-inositol 2-dehydrogenase	
lp_0505	0,56	*sdhB*	L-serine dehydratase, beta subunit	
lp_3606	0,57	*iolG2*	myo-inositol 2-dehydrogenase	
lp_3484	0,58	*lacM*	beta-galactosidase, small subunit	
lp_3314	0,58	*pflA2*	formate acetyltransferase activating enzyme	
lp_3607	0,58	*iolE*	inositol catabolism protein IolE	
lp_3313	0,59	*pflB2*	formate C-acetyltransferase	
lp_3608	0,60	*iolG3*	myo-inositol 2-dehydrogenase	
lp_1497	0,61	*narG*	nitrate reductase, alpha chain	
lp_1109	0,62	*citF*	citrate lyase, alpha chain	**Energy metabolism (10%)**
lp_1498	0,64	*narH*	nitrate reductase, beta chain	
lp_3483	0,64	*lacL*	beta-galactosidase, large subunit	
lp_2390	0,64	*bcaT*	branched-chain amino acid aminotransferase	
lp_2096	0,65	*fruK*	1-phosphofructokinase	
lp_0952	0,65		fumarate reductase, flavoprotein subunit precursor	
lp_0050	1,52	*pnb*	p-nitrobenzoate reductase	
lp_3420	1,60	*gadB*	glutamate decarboxylase	
lp_1112	1,63	*fum*	fumarate hydratase	
lp_0305	1,65	*gcsH1*	glycine cleavage system, H protein	
lp_0789[3]	1,83	*gapB*	glyceraldehyde 3-phosphate dehydrogenase	
lp_3045	1,85		short-chain dehydrogenase/oxidoreductase	
lp_0500	1,86	*rbsK1*	ribokinase	
lp_2629	5,02	*pox3*	pyruvate oxidase	
lp_3589	9,39	*pox5*	pyruvate oxidase	
lp_1672	0,28	*acpA2*	acyl carrier protein	
lp_1670	0,32	*fabZ1*	(3R)-hydroxymyristoyl-[acyl carrier protein] dehydratase	
lp_1673	0,32	*fabD*	[acyl-carrier protein] S-malonyltransferase	
lp_1674	0,33	*fabG1*	3-oxoacyl-[acyl-carrier protein] reductase	
lp_1675	0,33	*fabF*	3-oxoacyl-[acyl-carrier protein] synthase II	
lp_1676	0,34	*accB2*	acetyl-CoA carboxylase, biotin carboxyl carrier protein	
lp_1678	0,35	*accC2*	acetyl-CoA carboxylase, biotin carboxylase subunit	**Fatty acid and phospholipid metabolism (5%)**
lp_1671	0,36	*fabH2*	3-oxoacyl-[acyl-carrier protein] synthase III	
lp_1680	0,37	*accA2*	acetyl-CoA carboxylase, carboxyl transferase subunit alpha	
lp_1679	0,38	*accD2*	acetyl-CoA carboxylase, carboxyl transferase subunit beta	
lp_1681	0,42	*fabI*	enoyl-[acyl-carrier protein] reductase (NADH)	
lp_1682	0,48		phosphopantetheinyltransferase	
lp_0371	2,01	*glpD*	glycerol-3-phosphate dehydrogenase	
lp_2095	0,61	*fruR*	transcription regulator of fructose operon	
lp_3655	0,63	*srlM2*	sorbitol operon activator	
lp_2651	0,64		transcription regulator	
lp_2256	0,65	*ccpA*	catabolite control protein A	
lp_3234	0,66		transcription regulator	
lp_0825	1,54		transcription regulator (putative)	Regulatory functions (4%)
lp_0126	1,57		stress-responsive transcription regulator (putative)	
lp_0294	1,60		transcription regulator (putative)	
lp_1360	3,31		transcription regulator	
lp_2063[2]	4,84	*lexA*	transcription repressor of the SOS regulon	
lp_1853[3]	1,75	*rnhB*	ribonuclease HII	Transcription (1%)
lp_2278	1,98	*rhe3*	ATP-dependent RNA helicase	

Locus	FC[2] treatment	Gene	Product	Main Functional Class (%)[1]
lp_0580	0,49		HD superfamily hydrolase	
lp_1081	0,52		unknown	
lp_2939[2]	0,53		unknown	
lp_1486	0,57		unknown	
lp_1485	0,58		unknown	
lp_1484[3]	0,59		unknown	
lp_1872	0,61		unknown	
lp_2669	0,62		unknown	
lp_0063	0,66		unknown	
lp_0899	0,66		unknown	
lp_0155	0,66		unknown	
lp_1637	1,51		unknown	
lp_2573	1,51		unknown	
lp_1163	1,51		nucleotide-binding protein, universal stress protein UspA family	
lp_1439	1,51		unknown	
lp_0053	1,52		unknown	
lp_3359	1,53		integral membrane protein	
lp_0030[2]	1,56		unknown	
lp_1723	1,57		hydrolase, HAD superfamily, Cof family	
lp_3438	1,57		unknown	
lp_2937	1,59		unknown	
lp_1704	1,59		integral membrane protein	
lp_0990	1,60		unknown	
lp_2337	1,61	*cshA3*	1 segregation helicase (putative)	
lp_1292	1,62		acetyltransferase (putative)	
lp_3448	1,63		unknown	
lp_1290	1,65		integral membrane protein	
lp_2513	1,66		unknown	
lp_0837	1,71		unknown	Hypothetical proteins (22%)
lp_2224[2]	1,72		unknown	
lp_1886	1,78		unknown	
lp_2631	1,78		lipase/esterase (putative)	
lp_2718	1,82		unknown	
lp_0533	1,86		integral membrane protein	
lp_0981[2]	1,89		integral membrane protein	
lp_0927	1,93		unknown	
lp_1703	1,97		unknown	
lp_3356	1,98		acetyltransferase (putative)	
lp_0306	2,08		unknown	
lp_0207[2]	2,09		unknown	
lp_0091[2]	2,15		unknown	
lp_2279	2,23		exopolyphosphatase-related protein (putative)	
lp_2212	2,25		unknown	
lp_0089	2,32		unknown	
lp_2342	2,37		unknown	
lp_1939	2,42		oxidoreductase	
lp_0928	2,49		unknown	
lp_0307	2,55		unknown	
lp_0499	2,66		unknown	
lp_1543	2,69		1 segregation helicase (putative)	
lp_0145[3]	2,73		unknown	
lp_2113	2,84		unknown	
lp_1880	2,87		unknown	
lp_3141[2]	3,03		unknown	
lp_1708	4,19		unknown	
lp_3142	4,71		unknown	
lp_3022[2]	5,30		unknown	
lp_0960	9,18		unknown	
lp_1611[2]	32,85		unknown	

Locus	FC[2] treatment	Gene	Product	Main Functional Class (%)[1]
lp_2442	0,47		prophage P2a protein 15	
lp_0650	0,48		prophage P1 protein 27	
lp_2432	0,49		prophage P2a protein 25	
lp_2433	0,49		prophage P2a protein 24	
lp_0643	0,50		prophage P1 protein 20	
lp_0653	0,50		prophage P1 protein 30	
lp_2430	0,51		prophage P2a protein 27	
lp_2431	0,51		prophage P2a protein 26	
lp_0648	0,52		prophage P1 protein 25	
lp_0647	0,53		prophage P1 protein 24	
lp_0641	0,53		prophage P1 protein 18	
lp_0640	0,53		prophage P1 protein 17	
lp_2428	0,54		prophage P2a protein 29	
lp_0649	0,55		prophage P1 protein 26	
lp_0654	0,55		prophage P1 protein 31	
lp_2441	0,55		prophage P2a protein 16	
lp_2427	0,56		prophage P2a protein 30	
lp_0638	0,56		prophage P1 protein 15	
lp_2429	0,58		prophage P2a protein 28	
lp_2437	0,58		prophage P2a protein 20	
lp_0642	0,58		prophage P1 protein 19	**Other categories (13%)**
lp_2440	0,60		prophage P2a protein 17	
lp_0655	0,61		prophage P1 protein 32	
lp_0679	0,61		prophage P1 protein 56	
lp_0637	0,61		prophage P1 protein 14	
lp_0652	0,61		prophage P1 protein 29	
lp_2444	0,63		prophage P2a protein 13	
lp_0636	0,63		prophage P1 protein 13	
lp_2446	0,63		prophage P2a protein 11	
lp_1192	0,63		transposase, fragment (putative)	
lp_0635	0,65		prophage P1 protein 12	
lp_2450	0,65		prophage P2a protein 7	
lp_2445	0,65		prophage P2a protein 12	
lp_1854	1,54		GTPase	
lp_2455	2,65		prophage P2a protein 2, integrase	
lp_0624	2,80		prophage P1 protein 1, integrase	
lp_2919	0,66	*pepR2*	prolyl aminopeptidase	
lp_0959	1,85	*pepD3*	dipeptidase	Protein fate (1%)
lp_1836	3,34	*msrA3*	protein-methionine-S-oxide reductase	
lp_0581	0,47	*npsB*	non-ribosomal peptide synthetase NpsB	
lp_0578	0,53	*npsA*	non-ribosomal peptide synthetase NpsA	
lp_0582	0,57	*npsC*	4'-phosphopantetheinyl transferase	
lp_1973	0,64	*rpsU*	ribosomal protein S21	
lp_1048	1,51	*rpsN*	ribosomal protein S14	
lp_1034	1,53	*rplD*	ribosomal protein L4	
lp_1255	1,55	*prfC*	peptide chain release factor 3	
lp_0009	1,55	*rpsF*	ribosomal protein S6	**Protein synthesis (6%)**
lp_1036	1,56	*rplB*	ribosomal protein L2	
lp_2216	1,62	*rpsN2*	ribosomal protein S14-2	
lp_1535a	1,74	*rpmF*	ribosomal protein L32	
lp_1035	1,76	*rplW*	ribosomal protein L23	
lp_2285	1,80	*queA*	S-adenosylmethionine tRNA ribosyltransferase-isomerase	
lp_0737	1,80		ribosomal protein S30EA	
lp_2119	1,82	*tuf*	elongation factor Tu	
lp_1077	1,90	*rplM*	ribosomal protein L13	

Locus	FC[2] treatment	Gene	Product	Main Functional Class (%)[1]
lp_2932	0,61	*nrdD*	anaerobic ribonucleoside-triphosphate reductase	
lp_0761	1,77	*trxB1*	thioredoxin reductase (NADPH)	
lp_2729	1,80	*purE*	phosphoribosylaminoimidazole carboxylase, catalytic subunit	
lp_1289	1,89		purine/pyrimidine phosphoribosyltransferase (putative)	
lp_2728	1,91	*purK1*	phosphoribosylaminoimidazole carboxylase, ATPase subunit	
lp_2723	2,18	*purF*	amidophosphoribosyltransferase precursor	
lp_2724	2,32	*purL*	phosphoribosylformylglycinamidine synthase II	
lp_2727	2,33	*purC*	phosphoribosylaminoimidazole-succinocarboxamide synthase	**Purines, pyrimidines, nucleosides and nucleotides (6%)**
lp_2725	2,35	*purQ*	phosphoribosylformylglycinamidine synthase I	
lp_0497	2,38	*deoC*	deoxyribose-phosphate aldolase	
lp_2726	2,42	*purS*	conserved purine biosynthesis cluster protein	
lp_2719	2,91	*purD*	phosphoribosylamine--glycine ligase	
lp_2721	3,06	*purN*	phosphoribosylglycinamide formyltransferase	
lp_2720	3,14	*purH*	bifunctional protein: phosphoribosylaminoimidazolecarboxamide formyltransferase; IMP cyclohydrolase	
lp_2722	3,44	*purM*	phosphoribosylformylglycinamidine cyclo-ligase	
lp_0577	0,53	*pts9D*	mannose PTS, EIID	
lp_0502	0,55	*sdaC*	serine transporter	
lp_0576	0,58	*pts9C*	mannose PTS, EIIC	
lp_1087	0,60	*lp_1087*	cation transport protein	
lp_2650	0,60	*pts19B*	N-acetylgalactosamine PTS, EIIB	
lp_3604	0,62		myo-inositol transport protein	
lp_2648	0,64	*pts19D*	N-acetylgalactosamine PTS, EIID	
lp_0575	0,64	*pts9AB*	mannose PTS, EIIAB	
lp_2649	0,65	*pts19C*	N-acetylgalactosamine PTS, EIIC	
lp_1475	0,65	*fecE*	iron chelatin ABC transporter, ATP-binding protein	
lp_2097	0,65	*pts16ABC*	fructose PTS, EIIABC	
lp_0982	1,51		amino acid transport protein	
lp_3285	1,52	*qacH*	quaternary ammonium compound-resistance protein	
lp_1335	1,52		ABC transporter, ATP-binding protein	
lp_2836	1,54		multidrug transport protein	
lp_0367	1,55	*choS*	glycine betaine/carnitine/choline ABC transporter, substrate binding and permease protein	
lp_1473	1,59	*fecB*	iron chelatin ABC transporter, substrate binding protein (putative)	Transport and binding proteinsm (13%)
lp_0729	1,61		transport protein	
lp_0230	1,62	*pts2CB*	mannitol PTS, EIICB	
lp_0991	1,62		multidrug transport protein	
lp_2823	1,64		ABC transporter, ATP-binding protein	
lp_3279	1,65	*kup2*	potassium uptake protein	
lp_1472	1,66		ABC transporter component, iron regulated (putative)	
lp_2822	1,71		ABC transporter, permease protein	
lp_2038	1,76		transport protein	
lp_2992	1,78	*mntH2*	manganese transport protein	
lp_1466	1,94	*feoB*	ferrous iron transport protein B	
lp_1468	1,96		ABC transporter, ATP-binding protein	
lp_1469	1,98		ABC transporter component (putative)	
lp_1610	2,01	*opuD*	glycine betaine/carnitine/choline ABC transporter, permease protein	
lp_3288	2,03		cation efflux protein	
lp_0331	2,17		sugar transport protein	
lp_1467	2,46	*feoA*	ferrous iron transport protein A	
lp_0498	2,91		fucose transport protein	
lp_p3_05	0,61			
lp_p1_01	0,66			Plasmids (1%)
lp_RNA02	3,17			

[1]values given in parenthesis correspond to the percentage of the total amount of genes (267) that belong to each depicted functional class.

[2] upstream region of gene contains the lexA-dinR regulatory motif: **AGAACTCATGTTCG**

[3] upstream region of gene contains an uncharacterized regulatory motif: **AGCTAATAGCATCGGC**
